# Individual differences in naturally occurring affect predict conceptual breadth: evidence for the importance of arousal by valence interactions

**DOI:** 10.1186/s41235-022-00447-w

**Published:** 2022-11-18

**Authors:** Andrew Chung, Michael A. Busseri, Karen M. Arnell

**Affiliations:** Department of Psychology, Brock University, 1812 Sir Isaac Brock Way, St. Catharines, ON L2S 3A1 Canada

**Keywords:** Affect, Conceptual breadth, Individual differences, Latent variable modeling

## Abstract

Several studies have investigated the effect of induced mood state on conceptual breadth (breadth and flexibility of thought). Early studies concluded that inducing a positive mood state broadened cognition, while inducing a negative mood state narrowed cognition. However, recent reports have suggested that valence and arousal can each influence conceptual breadth. Individual differences in affective dispositions may bias perceptions, thoughts, and behaviors and, in turn, may be biased by them. Here, we examine whether individual differences in valence and arousal dimensions of self-reported, naturally occurring affect relate to conceptual breadth (using the Remote Associates Test, the Object Categorization Task, and the Alternative Uses Task), with no mood manipulations or cues. The three conceptual breadth tasks loaded onto a latent conceptual breadth factor that was predicted significantly by the interaction of valence and arousal. For participants low in arousal, greater positive affect was associated with greater conceptual breadth. For participants high in arousal, greater positive affect was associated with reduced conceptual breadth. In contrast to most existing theories of conceptual breadth that highlight the importance of valence or arousal alone, the present results suggest that the interaction between arousal and valence is key to predicting individual differences in conceptual breadth. We posit that positive mood states predict greater conceptual breadth in the presence of low versus high arousal due to a relaxation of cognitive control under low arousal.

**Supplementary Information:**

The online version contains supplementary material available at 10.1186/s41235-022-00447-w.

## Significance

When we measure conceptual breadth, we measure how broad and flexible our thinking is. For example: How many different uses for a brick can you think of in 3 min?, Is a skateboard a vehicle?, What single word unites the words “family,” “apple,” and “house”? Our ability to think broadly and flexibly allows us to solve everyday problems, but individuals differ reliably in their conceptual breadth. Why do some individuals receive high scores on conceptual breadth tasks while others receive low scores? Are the individuals who score highly on one conceptual breadth task the same ones who score highly on other conceptual breadth tasks? When individuals are induced into certain mood states with stimuli such as emotional videos, their conceptual breadth scores change. This suggests a relationship between affect and conceptual breadth, and this relationship is examined here in terms of individual differences in positive and negative affect predicting individual differences in conceptual breadth. Individuals answered questions about their typical naturally occurring affect in daily life and performed three different conceptual breadth tasks of the sort described above. We observed that conceptual breadth (as reflected in the commonality among the three tasks) was predicted by individual differences in affect, specifically the interaction between valence and arousal. For individuals low in arousal, greater positive affect was associated with greater conceptual breadth. For participants high in arousal, greater positive affect was associated with reduced conceptual breadth.

## Introduction

Our mood state can influence how we process and attend to information. There is abundant evidence to suggest that induced mood states can modify the breadth of our perception, attention, and cognitions (cognitive breadth). Cognitive breadth can be broadly categorized into attentional/perceptual breadth (the tendency to concentrate attention locally in a small region of space versus allocating it more globally over a larger region), and conceptual breadth (defined here as breadth and flexibility of thought). Here, we will examine the nature of the relationship between naturally occurring affect and conceptual breadth.

Early studies of affect and conceptual breadth seemed to paint a clear and simple picture—namely that positive affect increases conceptual breadth. For example, induced positive mood results in a greater ability to find the common word uniting three seemingly disparate words on the Remote Associates Test (RAT; Bolte et al., 2003; Isen et al., 1987; Rowe et al.,. 2007). Participants induced into a positive mood state are also more likely to produce creative, novel solutions to problems (Isen et al., 1987), make more atypical word associations (Isen et al., 1985), and are more flexible and inclusive about assigning items to categories (Isen & Daubman, 1984; see Baas et al., 2008 for a meta-analysis of mood and creativity). Positive affect has also been shown to increase the use of a broad, “gist” processing style (Vanlessen et al., 2013, 2014), increase the use of stereotype information versus information about individual behaviors (Isbell, 2004), reduce the effort when evaluating or categorizing stimuli (e.g., Park & Binaji, 2000; Schwarz, 1990), increase priming for irrelevant information (Biss & Hasher, 2011), and decrease perseveration and increase distraction (Dreisbach & Goschke, 2004).

Such findings are consistent with Fredrickson’s “Broaden and Build” theory of positive emotions (e.g., Conway et al., 2013; Fredrickson, 2001; Fredrickson & Branigan, 2005), where positive affect is thought to reflect safety, such that it allows for more varied thought–action repertoires, allowing the organism to be flexible and open to exploring ideas and the broader environment. In contrast, negative affect sends a signal that attention and thought must be focused on the locus of potential threat. Relatedly, some models posit that different moods set different contexts for cognitive processing, where positive mood signals safety, thereby relaxing cognitive processing so that a loose, gist style is preferred, whereas negative mood signals a problematic state that should be rectified with careful systematic processing (e.g., Schwarz, 1990).

However, positive affect has not always been shown to broaden cognition when it was predicted to do so (e.g., Bruyneel et al., 2013; Fan, et al., 2002). Also, Huntsinger et al. (2010) showed that positive affect strengthened whatever breadth level was dominant given the circumstances. Huntsinger and Ray (2016) explained these results in terms of happy mood reinforcing the dominant context, while a negative mood suggested a change from the dominant context was needed (see also Hunsinger et al., 2012).

As noted by Gable and Harmon-Jones (2008), negative affect is often induced using negative material that is high in both arousal and motivational intensity, invoking states such as fear, disgust, and anxiety (e.g., presentation of gory scenes, negative-arousing video clips, or inducing performance stress). In contrast, positive affect is often induced using positive materials low in both arousal and motivational intensity, invoking states such as amusement or calmness (e.g., humorous videos, cute pictures, or recalling a pleasant event). Therefore, the confounding of emotional valence (the positive to negative subjective evaluation of a stimulus) with arousal (the level of activation produced by the stimulus) and motivational intensity (the urge to move toward/away from a stimulus as opposed to a lack of motivation for movement) makes it unclear whether the positive valence, low arousal, low motivational intensity, or the combination of these factors lead to the increase in cognitive breadth shown in many of the above-described studies. Indeed, approach-motivated drive and high arousal have been linked to reduced breadth of cue utilization in both humans (e.g., Bahrick et al., 1954) and animals (e.g., Bruner et al., 1955). And as early as 1959, Easterbrook proposed a model of drive and cue utilization which stated that during a high state of arousal, the range of cues that an individual will use is severely restricted, resulting in an increase in the likelihood of a dominant response.

In a series of studies, Harmon-Jones, Gable, and colleagues have provided compelling evidence that breadth of cognition can be influenced by motivational intensity, such that affective states that are low in motivational intensity result in an increase in attentional and conceptual breadth, whereas high motivational intensity states result in a decrease in attentional and conceptual breadth (see Gable & Harmon-Jones, 2010a, 2010b; Harmon-Jones et al., 2012 for reviews). For example, Price and Harmon-Jones (2010) demonstrated the impact of motivational intensity on conceptual breadth when they showed that participants classified items in terms of narrower, less inclusive categories when leaning forward and smiling (high positive motivational intensity as in desire), relative to when sitting upright and smiling. However, they classified items in terms of broader, more inclusive, categories when reclining backward and smiling (low positive motivational intensity as in relaxation).

Some models state that cognitive breadth depends on the interaction of arousal, valence, and motivational intensity. For example, in their activated–orientation hypothesis, Baas et al. (2008) posit that for deactivated (low arousal) states, the motivational intensity will not modify the cognitive breadth. In contrast, according to the authors, at higher levels of arousal, approach states should increase cognitive breadth, but avoidance states should decrease it.

Gasper and Middlewood (2014) examined the interaction of valence, arousal, and motivational direction (approach versus avoidance), using film clips to induce participants into one of four affective states: elation (positive, activated, approach), distress (negative, activated, avoidance), relaxation (positive, deactivated, avoidance), and boredom (negative, deactivated, approach). Participants correctly solved more questions on the RAT and were more flexible and inclusive in terms of assigning items to categories, when they were elated or bored, relative to when they were relaxed or distressed. This led the authors to conclude that approach-oriented states facilitated conceptual breadth relative to avoidance states. However, the film clips used to induce elation (the orgasm scene from the movie When Harry Met Sally) and boredom (a silent screensaver with moving, colored sticks) both seem unlikely to induce motivationally intense approach states.

### Naturally occurring self-report measures of affect

As seen from the literature reviewed above, studies investigating conceptual breadth and emotion have typically induced mood states in participants and then compared the patterns of conceptual breadth across groups. This approach has many advantages, including in terms of making cause-and-effect claims. However, as noted above, even with mood manipulation checks, it is sometimes difficult to know what mood is being induced with specific film clips and how that may interact with the affective dispositions or current mood state of the participant. As such, one may wonder whether the patterns observed with induced mood can also be found based on individual differences in naturally occurring affect. That is, are individuals who report positive and/or low arousal affective dispositions more likely to show a broader cognitive scope than individuals who report negative and/or high arousal affective dispositions? This next step is important, as it is possible that affect only influences cognitive breadth due to a phasic response evoked by an environmental trigger, such as the affective videos used to induce affective states or pictures used as cues. Naturally occurring affect may influence an individual’s typical or default breadth of cognition, and this in turn could influence their performance on several conceptual breadth tasks under conditions where mood is not manipulated. Typical affect could promote habitual broad or narrow processing which could then promote particular affective states that then reinforce particular processing modes in a reciprocal relationship. Indeed, Bar (2009) posits that narrow activation of associations reinforces depressive rumination, which in turn promotes narrow activation.

Only a few studies have looked at whether individual differences in naturally occurring state affect are associated with conceptual breadth. Isbell (2004) showed that individuals who reported being happier showed greater use of generalized trait stereotype information, as opposed to using specific behaviors, when forming an impression of an individual described in a vignette. However, Hunsinger et al. (2012) showed that this effect reversed when happy individuals were primed by a local context versus a global context. Gasper and Middlewood (2014) asked participants to self-report their current levels of elation, boredom, distress, and relaxation and showed more inclusiveness and flexibility in categories for individuals high in approach versus avoidance states (elation and boredom minus relaxation and distress). Middlewood et al. (2016) showed that self-reported state of tiredness and happiness interacted to predict greater acceptance of atypical ideas on a category rating task and on a gestalt completion task where people had to identify abstract images. Specifically, being both tired and happy led to the greatest acceptance of atypical ideas reflective of conceptual breadth, suggesting an interaction of valence and arousal. Therefore, the small literature thus far provides preliminary evidence that the interaction of arousal and valence may be particularly important for understanding the association between affect and conceptual breadth.

### The present study

The goal of the present study is to examine whether individual differences in self-reported naturally occurring affect can predict individual differences in conceptual breadth and, if so, whether affective valence, arousal, or the interaction of valence and arousal are significant predictors of conceptual breadth. This is a novel contribution as thus far only two studies (Gasper & Middlewood, 2014; Middlewood et al., 2016) have examined whether self-reported naturally occurring affect is related to conceptual breadth with mood states varying in both arousal and valence, and both examined discrete mood states as opposed to testing the interaction of valence and arousal dimensions. In the present study, affect was measured with items from the Circumplex Affect Questionnaire (Feldman-Barrett & Russell, 1998) which are based on Russell’s circumplex affect model (Feldman-Barrett & Russell, 1998; Russell, 1980; Russell & Barrett, 1999). According to this model, there are two orthogonal dimensions to affect: valence (positive to negative) and arousal (deactivated to activated). As such, the questions on the Circumplex Affect Questionnaire were made to reflect low or high arousal at various degrees of positive/negative valence, and positive or negative valence at various levels of arousal. Therefore, this instrument allowed us to examine whether conceptual breadth could be predicted by the activation–deactivation dimension (subsequently referred to here as arousal), the positive–negative valence dimension, and/or the interaction of these two dimensions.

In this study, conceptual breadth was assessed with three varied tasks: the Remote Associates Task (RAT; Mednick & Mednick, 1971), an Object Categorization Task that asks participants whether various exemplars belong in a specific category (Isen & Daubman, 1984), and the Alternative Uses Task (Guilford, 1956). Because such varied tasks were used, conceptual breadth here reflects broad and flexible thought, as opposed to divergent thinking or creativity per se. An individual’s score on any one task is a combination of measurement error, domain-specific processes, domain-general executive processes (e.g., Kovacs & Conway, 2016), and variance that is specific to the stimuli, task, use of the response scale, or the response method employed, in addition to the measurement of the actual underlying construct. This can result in attenuated correlations between scores on various tasks purporting to measure the same construct and between these scores and other predictors (e.g., Schmiedek et al., 2009). Here, we use a latent variable modeling approach, which has not been used previously in studies of conceptual breadth and affect by creating a latent factor representing the variability shared by the three conceptual breadth tasks in order to minimize the noise and task-specific variability that potentially underestimates the strength of the relationships so that we can better assess the relationship between naturally occurring affect and conceptual breadth. Of course, this latent variable could also include variability due to domain-general executive processes (e.g., Kovacs & Conway, 2016), but much of the task-specific, domain-specific and random error will be removed.

Given the wealth of studies showing that induced low arousal positive affect results in increased conceptual breadth, we predict that conceptual breadth will increase with positive valence for those low in arousal. Given the evidence of Gable, Harmon-Jones, and colleagues, we predict that conceptual breadth will decrease with positive valence for those high in arousal.

## Methods

### Participants

The 139 participants were undergraduate students from Brock University between the ages of 18 and 38 years (*M* = 19.8 years; 88% female). All participants reported normal or corrected to normal vision and learning English before the age of 8 years. Participants received bonus research participation credit toward a course. The stopping rule was to run as many participants as possible before the end of the term when research participation expired in an Introductory Psychology course. One participant gave RAT responses that suggested they did not understand the task, and another participant recorded words incorrectly in the object categorization task, so these data were not scored. Participants completed the affect questionnaire first, before the RAT, followed by the Object Categorization Task, and lastly the Alternative Uses Task. Only participants who completed all of the conceptual breadth measures and the affect questionnaire were included in the analyses (6 were incomplete). As a result, 131 participants remained in the sample. There were no missing values on the affect measures or the cognitive tasks for these participants.

A post hoc sensitivity analysis conducted using GPower indicated that, for simple linear correlations and simple slopes (when dissecting the valence by arousal interaction), with a two-tailed test, *α* = 0.05, and *N* = 131, using Cohen’s (1988) norms for small (*r* = 0.10), medium (*r* = 0.30) and large (*r* = 0.50) effects, the observed power was 0.21, 0.94 and 0.99, respectively. Further, for bivariate correlations there was 80% power to find relationships of *r* = 0.241 or larger. The main analysis examined whether valence, arousal, and the interaction of valence and arousal could predict the latent conceptual breadth factor. Sensitivity analysis for *R*^2^ increase in a multiple linear regression with *α* = 0.05, *N* = 131, and 3 tested predictors showed there was 80% power to find unique predictor effects (partial *r*^2^) = 0.085 or larger.

### Measures

#### Affect

The Circumplex Affect Questionnaire (Feldman-Barrett & Russell, 1998) contains 77 phrases such as “I’m filled with energy” and “I feel disturbed and upset” (the statements from Sects.  1 and 2 from Studies 2 and 3 of Feldman-Barrett & Russell, 1998). Participants used a 5-point Likert scale to indicate the degree to which they found each statement to be true in terms of how well it described them generally in their everyday life over the past several weeks (1 = strongly disagree, 5 = strongly agree). Participants were encouraged to answer based on how they typically felt, not how they felt in the moment, or how they hope to feel in the future. We specifically asked participants to report their typical trait affect, not their current state affect, given that individual differences in cognitive breadth have been shown to be relatively stable over time (e.g., Dale & Arnell, 2013). Further, we were working from the idea that typical affect could promote habitual broad or narrow processing, which could then promote particular affective states, that then reinforce particular processing modes in a reciprocal relationship (e.g., Bar, 2009). The questionnaire contains questions that assess activation and deactivation at pleasant, neutral, and unpleasant valence, and pleasant and unpleasant valence at high, medium, and low activation. For each participant, an overall arousal score was calculated as the difference between their average ratings for the 38 activation items (Cronbach’s *α* = 0.837 here) and the 28 deactivation items (Cronbach’s *α* = 0.684 here). Similarly, an overall valence score was calculated as the difference between their average ratings for the 32 pleasant items (Cronbach’s *α* = 0.951 here) and the 33 unpleasant items (Cronbach’s *α* = 0.936 here).

### Conceptual breadth measures

#### RAT

Participants completed the RAT (Mednick & Mednick, 1971), which consists of twenty questions listed vertically on a single piece of paper. For each question there were three key words placed horizontally, followed by a blank box where the participant was asked to write the word that was common to the three key words (for example, the correct answer to the question with APPLE, FAMILY, and HOUSE would be TREE). Participants were told that they had 10 min to get as many correct answers as possible and that they could go through the questions in any order and come back to questions at any time. Participants were given a practice question with its correct answer prior to starting. The number of correct responses out of 20 was used as a participant’s RAT score. The RAT has been shown to have good internal reliability (Cronbach’s *α* = 0.82) and has been shown to be a stable measure (Spearman Brown reliability = 0.92 (Lee et al., 2014).

#### Object categorization task (OCT)

Participants completed the Object Categorization Task previously used by Isen and Daubman (1984) and Price and Harmon-Jones (2010) to measure cognitive organization. Word categories and exemplars were chosen from those developed by Rosch (1975). Participants were asked to rate the degree to which they felt each exemplar fit into the specified category using a 10-point Likert scale (1 = not at all, to 10 = definitely belongs and is a great example of the category). For example, participants rated how well “chair” fits into the category of “furniture.” For this task, three categories (furniture, vehicles, and clothing) were shown in a fixed order, with nine exemplars for each category. For each category, three were strong exemplars of the category (for example, CHAIR, for furniture), three were moderately good exemplars (for example, BENCH for furniture), and three were weak exemplars (for example, VASE for furniture), based on findings from Rosch (1975). The first exemplar for each category was always strong, but the remaining exemplars were randomized and counterbalanced using a Latin Square for the order of presentation. For each exemplar, the experimenter read aloud the category name and the exemplar, and then each participant wrote the exemplar name and their rating. For each participant, mean category inclusion ratings were computed separately for the strong, moderate, and weak exemplars. Here, the Cronbach’s alpha = 0.617 for weak and moderate exemplars from three object categories (i.e., for the six OCT measures).

#### Alternative uses task (AUT)

We employed a task constructed by Guilford (1956) for the purpose of measuring divergent thinking. In this task, participants had four minutes to write down as many plausible uses they could think of for a common brick (e.g., a brick could be a pencil holder or used to build a step but could not be a remote control). For each participant, flexibility scores were calculated as the number of different use categories reported. For example, using a brick to build a house, construct a fence, and make a chimney would all fall under the same category of “construction.” The use of flexibility scores is a normative way of scoring the Alternative Uses Task (Gilhooly et al., 2007). The Alternative Uses task has been shown to have moderately good internal reliability (Cronbach’s *α* = 0.61) across exemplars (e.g., Tanis, 2017).

### Analysis plan

The main analyses first used bivariate correlations to examine the variability shared among each pair of conceptual breadth tasks. Because significant correlations were observed among conceptual breadth tasks, latent variable modeling was used to estimate a latent conceptual breadth factor. This allowed examination of whether or not all three conceptual breadth tasks would load on the latent measure. To do so, we estimated a measurement model in which the three cognitive breadth tasks (i.e., Remote Associates Test, Object Categorization Task, and Alternative Uses Task) were specified as loading onto a single latent factor. All three conceptual breadth scores were standardized prior to analysis using z-scores. All three loadings were freely estimated and the variance in the latent factor was fixed to 1.0 for identification purposes. See Additional file 1: Fig. S1 for additional model estimation details. This model and the one described below were computed using Mplus software (version 8) based on maximum likelihood (ML) estimation.

This latent factor representing the common latent variability in conceptual breadth also provided the dependent variable when we predicted conceptual breadth with the affect measures. Valence and arousal rating scores were standardized as z-scores and then the product of these served as the valence X arousal interaction. Structural equation modeling (SEM) was then used to examine valence, arousal, and the interaction between valence and arousal as simultaneous predictors of the latent conceptual breadth factor. In this model, the paths from valence, arousal, and the interaction term were each freely estimated, as were their correlations, along with the loadings of the three conceptual breadth tasks on the latent factor; the residual variance in the latent factor was fixed to 1.0 for identification purposes. See Additional file 1: Fig. S2 for additional model estimation details.

To dissect the significant interaction, a follow-up Johnson-Neyman technique was performed to examine the effect of valence at various levels of arousal (varying from − 3 to + 3 SDs). In each case, an alpha level cutoff of *p* < 0.05 was implemented to differentiate between significant and non-statistically significant findings.

To examine how robust the pattern of results was across various conceptual breadth tasks, and facilitate comparisons with previous studies, supplementary analyses were also performed to examine whether affect showed the same relationship to conceptual breadth for each conceptual breadth task individually and for all pairs of tasks. An additional analysis examined whether the same pattern of results was observed when a more common simultaneous regression approach was used in which conceptual breadth was operationalized as a composite score formed by averaging the z-scores for the three conceptual breadth tasks (Cronbach’s *α* = 0.50), as opposed to the SEM approach used in the main analysis based on a latent conceptual breadth factor.

## Results

### Preliminary analysis

#### Conceptual breadth measures

For the categorization task, a one-way repeated measures ANOVA comparing the mean ratings in the object categorization task as a function of the exemplar category strength (weak, moderate, and strong) was significant, *F* (2260) = 2337.36, *p* < 0.001, *η*^2^ = 0.947. Subsequent paired-samples *t* tests showed that weak exemplars (*M* = 3.67, *SD* = 1.15) were given significantly lower inclusion ratings than moderate exemplars (*M* = 8.03, *SD* = 0.87), *t*(130) = 42.10, *p* < 0.001; moderate exemplars were rated significantly lower than strong exemplars (*M* = 9.95, *SD* = 0.13), *t*(130) = 61.93, *p* < 0.001; and weak exemplars were rated significantly lower than strong exemplars, *t*(130) = 25.79, *p* < 0.001. However, a ceiling effect was observed for strong exemplars where almost all participants rated all strong exemplars a 10/10. Therefore, prior to subsequent analysis, an overall category inclusion rating score was calculated for each participant based on their average ratings for the weak and moderate (but not strong) exemplar categories.

### Primary analysis

All of the conceptual breadth measures showed a good range of individual differences (see Table 1). There were significant, yet modest correlations among the three conceptual breadth tasks (see top left triangle area in Table 2).

**Table 1:** Descriptive statistics for all measures

Measures	Mean	SD	Min	Max
RAT	7.42	3.40	0	17
OCT	5.85	0.83	3.50	7.78
AUT	7.10	2.79	1	19
Pleasant	3.19	0.59	1.44	4.72
Unpleasant	2.24	0.60	1.03	4.39
Valence (P-U)	0.95	1.10	− 2.96	3.69
Activation	2.60	0.37	1.61	3.45
Deactivation	2.82	0.33	2.04	3.50
Arousal (A-D)	− 0.22	0.51	− 1.71	1.32

*RAT* Remote Associates Test. *OCT* Object Categorization Task. *AUT* Alternative Uses Task. *P-U* pleasant minus unpleasant. *A-D* activation minus deactivation

**Table 2:** Correlations among conceptual breadth measures and affect measures

Measures	RAT	OCT	AUT	Valence	Arousal
RAT	1				
OCT	0.22*	1			
AUT	0.31**	0.22*	**1**		
Valence	0.08	− 0.10	− 0.00	1	
Arousal	0.00	0.05	0.00	0.19*	1

*Correlation is significant at the 0.05 level (2- tailed). **Correlation is significant at the 0.01 level (2-tailed). *RAT refers* Remote Associates Test. *OCT* Object Categorization Task. *AUT* Alternative Uses Task. Bold values shows correlations among the conceptual breadth tasks

#### Affect

Fairly large individual differences were observed in self-reported naturally occurring affect (see Table 1). The valence and arousal dimensions showed good reliability (both *α*s = 0.77). However, note that participants were generally positive overall so mean Pleasant minus Unpleasant Valence scores approximated 1. With a standard deviation of about 1 this means that participants with − 1 standard deviation on valence showed neutral valence, not a preponderance of negative valence. This was not true for the arousal dimension where mean Activated minus Deactivated scores approximated 0. Correlations among affect measures are reported in Table 2.

### Measuring conceptual breadth

**Fig. 1:**
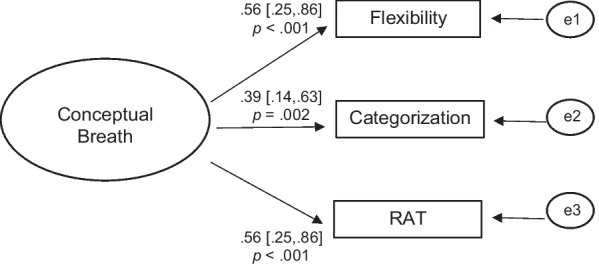
Results from the measurement model for the latent cognitive breadth factor. Note. The large oval represents the latent conceptual breadth factor; small ovals represent residual error variances. Rectangles are measured variables. Flexibility = Alternative Uses Task; Categorization = Object Categorization Task; RAT = Remote Associates Test. Standardized parameters are shown, along 95% confidence intervals and *p* values

The measurement model was saturated (i.e., *df* = 0) and thus provided perfect fit: model *χ*^2^ = 0; CFI = 1.00; SRMR = 0; RMSEA = 0; AIC = 1107.24. As shown in Fig. 1, all three conceptual breadth tasks had statistically significant and moderate loadings on the latent conceptual breadth variable. Indicator reliability values were as follows: 0.31 (flexibility), 0.15 (categorization), and 0.31 (RAT); average variance explained in the indicators = 0.26; composite reliability for the latent factor was 0.51. Together, these findings suggest that there was a significant, albeit moderate, amount of shared variance among the three cognitive breadth tasks. Further, the reliability of the latent factor as an overall indicator of conceptual breadth (as measured by the three tasks) was also moderate.

### Predicting conceptual breadth

#### Valence by arousal

The model provided excellent fit: model *χ*^2^ = 5.87, *df* = 6, *p* = 0.44; CFI > 0.99; SRMR = 0.05; RMSEA < 0.01, *p* for close fit = 0.63; AIC = 2213.53.

**Fig. 2:**
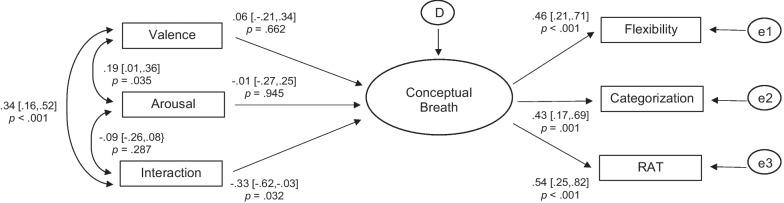
Results from the structural equation model predicting the latent cognitive breadth factor using affect valence, arousal, and their interaction. Note. The large oval represents the latent conceptual breadth factor; small ovals represent residual error variances (“e”) or the regression residual (disturbance, “D”). Rectangles are measured variables. Flexibility = Alternative Uses Task; Categorization = Object Categorization Task; RAT = Remote Associates Test. Standardized parameters are shown, along 95% confidence intervals and *p* values

As shown in Fig. 2, the latent conceptual breadth factor had statistically significant and moderate loadings from all three cognitive tasks (indicator reliability values = 0.21, 0.18, and 0.29 for flexibility, categorization, and RAT, respectively; average explained variance = 0.23; composite reliability = 0.47). Further, whereas the standardized path coefficients from affect valence and arousal were small and did not differ significantly from zero, the interaction of valence and arousal had a moderate and statistically significant unique predictive effect on the latent cognitive breadth factor (see Fig. 2 for details). This model explained 8.7% of the variance in the latent cognitive breadth factor.

**Fig. 3:**
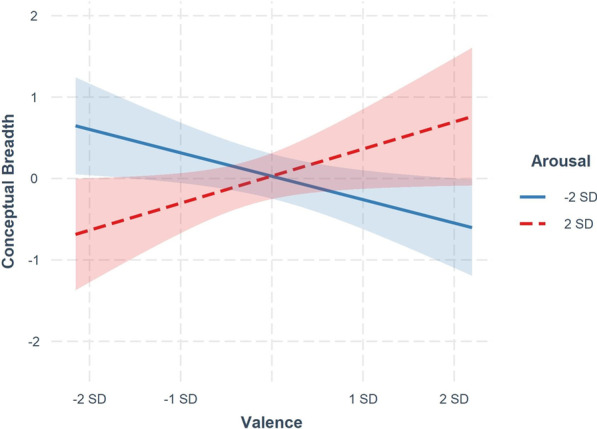
Simple slopes depicting the relationship between conceptual breadth and valence scores separately for those low or high in arousal. Note that participants were generally positive in valence overall, and as such − 1 SD for valence represents neutral valence as opposed to negative valence. Arousal showed a full range of scores from very deactivated to very activated. Shaded areas represent the 95% confidence intervals for each slope

Follow-up analysis based on the Johnson-Neyman technique revealed that at lower levels of arousal, the predictive effect of affect valence on cognitive breadth was positive in direction (e.g., at 2 SD below the mean for arousal, the simple slope for valence = 0.71, *p* = 0.055); in contrast, at higher levels of arousal, the predictive effect of affect valence was negative in direction (e.g., at 2 SD above the mean for arousal, the simple slope for valence = -0.59, *p* = 0.045). As illustrated in Fig. 3, these findings suggest that cognitive breadth increased as valence moved from neutral to positive for those with low arousal, whereas cognitive breadth decreased as valence moved from neutral to positive for those with high arousal.

### Supplementary analyses

#### Robustness across the various conceptual breadth tasks

One might wonder whether the pattern for the interaction effect depended primarily on the one of the three tasks, or whether this interaction pattern was robust for each paired combination of conceptual breadth measures, or even for each individual measure. Indeed, almost all previous work examining relationships between affect and conceptual breadth has used a single conceptual breadth task, so additional analyses are desirable to facilitate comparisons across studies.

To investigate this question, composite variables were first formed from each possible paired combination of two tasks from the three conceptual breadth measures (i.e., OCT and RAT, OCT and AUT, RAT and AUT) and individually subjected to the same regression analysis that was used for the three-measure composite variable. As before, the valence path and the interaction path never approached significance in any of the analyses (all *p*’s > 0.42), but the interaction path did. Very similar slope patterns were observed for each pair of tasks although the magnitude of the interaction effect varied somewhat (OCT and RAT together: Valence × Arousal path = − 0.46, *p* = 0.037, OCT and AUT together: Valence × Arousal path = − 0.34, *p* = 0.06, RAT and AUT together: Valence × Arousal path = − 0.22, *p* = 0.13).

To investigate the role of affect for each of the conceptual breadth tasks individually, three simultaneous regressions were used where valence, arousal, and their interaction were used to predict performance on the individual breadth task. (There was no need for SEM or a latent measure given that only one task was used as the criterion variable.) The main effects of valence and arousal did not predict performance on any of the three breadth tasks (all *p*’s > 0.40). However, all three conceptual breadth measures showed a similar interaction pattern where breadth decreased with positive affect at high arousal and increased with positive affect at lower arousal. This Valence by Arousal interaction was strongest and statistically significant for the OCT task (*β* = − 0.24 *p* = 0.011), marginally significant for the RAT task (*β* = − 0.18 *p* = 0.059), and nonsignificant with the flexibility scores from the AUT task (*β* = − 0.06 *p* = 0.57). With alpha correction using the Bonferroni method, the OCT results remain significant. These results show that while the same valence by arousal interaction pattern is numerically present for each of the conceptual breadth tasks, harnessing the common variability among conceptual breadth tasks using SEM allows for a more parsimonious and powerful test of the pattern.

### Measuring conceptual breadth

But is an SEM analysis necessary here or could a simple average of the standardized conceptual breadth scores from each task also show the same significant interaction effect? When valence, arousal and their interaction were used in a simultaneous regression to predict a composite average of the z-scores from the three conceptual breadth measures, a very similar pattern of results was observed. Specifically, both valence (*β* = 0.02, *p* = 0.64) and arousal (*β* = − 0.003, *p* = 0.76) were non-significant predictors, whereas the valence x arousal interaction (*β* = − 0.15 *p* = 0.018) reached significance with the same crossover interaction pattern observed in the SEM analysis. Follow-up analysis based on the Johnson-Neyman technique revealed that at -1.96 SDs or lower levels of arousal, the predictive effect of affect valence on conceptual breadth was significantly positive in direction (*p*’s < 0.05). In contrast, at + 1.18 SDs or higher levels of arousal, the predictive effect of affect valence was significantly negative in direction (*p’s* < 0.05). Therefore, both the SEM analysis and the regression analyses lead to the same conclusions.

## General discussion

The current study investigated whether individual differences in naturally occurring affect could predict conceptual breadth using a latent variable modeling approach. Participants’ self-reports of their naturally occurring affect significantly predicted individual differences in our latent conceptual breadth factor comprised of RAT scores (Mednick & Mednick, 1971), Object Categorization scores (Isen & Daubman, 1984), and scores from the Alternative Uses Task (Guildford, 1956). The interaction pattern showed that the predictive effect of valence was opposite in direction for individuals characterized by high versus low arousal. In particular, higher positive valence predicted greater conceptual breadth for those lower in arousal but predicted lower conceptual breadth for those higher in arousal.

### Implications for models of conceptual breadth

An important and novel finding was that substantial common variance was observed among all three different conceptual breadth measures, providing evidence for a unified concept of conceptual breadth across these tasks. In particular, all three tasks had moderate loadings on the latent conceptual breadth factor. This is noteworthy as attentional breadth measures such as those using Navon letters and hierarchical shapes have not been found to share common variability with each other, despite being quite highly reliable (Dale & Arnell, 2013). Of course, some of the shared variability captured by the latent conceptual factor may represent domain-general executive processes (e.g., Kovacs & Conway, 2016) in addition to conceptual breadth. However, as noted just above, attentional breadth tasks have not shown common variability even though such domain-general processes would likely be present there as well. Because the conceptual breadth tasks used here do share common variability, latent variable modeling may be useful for the *conceptual* breadth literature even though the Dale and Arnell (2013) paper suggests it may not be useful for the *attentional* breadth literature.

Previous studies investigating affect and conceptual breadth have examined the role of affect in predicting variability in individual breadth tasks but have not examined the common variability among conceptual breadth tasks, even when more than one breadth task was included in the study. This may be because the scores on these tasks are typically only modesty positively correlated with each other (as observed here). However, an individual’s score on any one task reflects other sources of variability in addition to the measurement of the actual underlying construct, and this results in attenuated correlations between scores on various tasks purporting to measure the same construct, and between these scores and other predictors (e.g., Schmiedek et al., 2009). For example, in the working memory literature, correlations between various working memory tasks are typically small which led some researchers to conclude these various tasks were measuring different constructs or at least different aspects of working memory (e.g., Redick & Lindsey, 2013). However, latent variable modeling has shown that these various working memory measures actually share substantial variability once task/stimulus/response specific noise and measurement error are removed and that this variability can be predicted by other variables such as reasoning (e.g., Schmiedek et al., 2009, 2014; Waris et al., 2017; Wilhelm et al., 2013).

As in the working memory literature, some researchers have suggested that conceptual breadth tasks are not well correlated with each other and as such may represent different aspects of conceptual breadth. For example, Lee et al. (2014) showed that there was a nonsignificant relationship between the Remote Associates Test and Guilford’s Unusual Uses Task and posited that the former should be considered a measure of convergent thinking whereas the latter should be considered a measure of divergent thinking. In contrast, the current results show that the three popular conceptual breadth tasks used in the current study load significantly on the same latent factor, and that this common variability can be predicted by the interaction between affect valence and arousal, but not by arousal or valence alone. Nonetheless, loadings on the latent factor were moderate, rather than large, in magnitude. Such findings are consistent with previous studies indicating modest correlations among cognitive tasks thought to reflect the same underlying latent ability or process (e.g., see Schmiedek et al., 2009, 2014 for working memory measures and Friedman & Miyake, 2017 for executive function measures). However, the moderate reliability of the latent conceptual breadth factor likely attenuated its associations with the other variables of interest, suggesting that the present findings concerning the estimated effects of affect valence, arousal, and their interaction may be conservative. In future studies, therefore, it would be useful to study conceptual breadth as a latent factor indicated by multiple indicators that were more strongly intercorrelated. Future conceptual breadth studies could also benefit from measuring performance on more than one conceptual breadth task and then examining the relationships with, or effects of, manipulations on the latent conceptual breadth score as opposed to only examining effects on a particular breadth task or dividing conceptual breadth tasks into subcategories.

What is the shared variability in the latent variable representing? We have labeled it conceptual breadth (defined here simply as breadth and flexibility of thought). We chose this label purposely because it is relatively broad and generic, and at present there is no ability to determine its nature more specifically. The three conceptual breadth tasks we chose were selected to be broad and tap various aspects such as breadth, inclusiveness, flexibility, and creativity. Latent variable labels such as creativity and divergent thinking seemed too specific and not well captured across all three tasks. For example, creativity would seem to be an important part of AUT flexibility scores and probably even RAT scores, but it is less clear how creativity would influence OCT scores. Indeed, the AUT flexibility measure was the one that had the weakest relationship with the valence by arousal interaction and the OCT was the one that had the strongest, suggesting the relationship between conceptual breadth and affect may be less about creativity and more about breadth and flexibility of thought. However, creativity may also contribute to this relationship and could be examined in future work.

Future studies could also include other tasks to help discover what aspects of conceptual breadth tasks might support the valence by arousal interaction. For example, creative problem-solving tasks such as the candle task (Isen et al., 1987), word association tasks (Isen et al., 1985) or tasks that tap the use of heuristics (e.g., Frederick, 2005; Park & Banaji, 2000) could also be included in the task set. Future studies could also include tasks tapping domain-general executive processes that do not include a conceptual breadth component along with conceptual breadth tasks to help isolate variability due to these domain-general processes versus conceptual breadth.

### Implications for models of affect and conceptual breadth

In contrast to most existing theories of conceptual breadth that highlight the importance of valence or arousal alone, the present results suggest that the interaction between arousal and valence is key to predicting individual differences in conceptual breadth. This has implications for understanding conceptual breadth by informing us about the conditions under which conceptual breadth might be strongest (i.e., under conditions of low arousal positive affect). The present results also have implications for theoretical models of conceptual breadth. The finding of greater conceptual breadth among those lower in arousal and higher in positive affect is somewhat consistent with valence models such as Broaden and Build (Fredrickson, 2001) that link greater conceptual breadth to greater positive affect. The only caveat is that this is observed for low arousal states only. The present results also underscore Harmon-Jones and colleagues’ (e.g., Gable & Harmon-Jones, 2010a, 2010b; Harmon-Jones et al., 2012) point that dimensions other than affect valence can moderate how affect influences cognitive breadth. Specifically, the present finding that greater positive affect is associated with reduced attentional breadth under conditions of high arousal is consistent with their findings of reduced breadth for stimuli evoking desire.

The pattern of results reported here can also explain some previous inconsistencies in the literature on affect and cognitive breadth and explain why there is evidence in the literature for both valence and arousal predicting conceptual breadth. As noted above, early studies of affect and cognitive breadth often confounded arousal and valence by inducing high arousal negative affect and low arousal positive affect, but then attributed the greater breadth observed in the low arousal positive condition to the effect of positive valence. When examining only low arousal, the effects of valence predicted by models such as Broaden and Build (Fredrickson, 2001) can be observed—namely the pattern observed here where a positive relationship between positive affect and cognitive breadth is observed at lower levels of arousal. However, when examining only high arousal, the findings that desire narrows attention reported by Harmon-Jones and colleagues (e.g., Gable & Harmon-Jones, 2008) can be explained by the pattern observed here where a negative relationship between positive affect and cognitive breadth is observed at higher levels of arousal. Similarly, Lacey et al. (2021) recently argued that the interaction of motivational intensity and valence is key to understanding cognitive breadth. Future research should examine whether motivational intensity and arousal fulfill a similar role with respect to interacting with valence to predict conceptual breadth.

The present results are also consistent with those of Middlewood et al. (2016) who showed that the interaction of self-reported tired (a low arousal state) and happy (a positive state) predicted more acceptance of atypical exemplars in the category ratings task, and more wrong but plausible guesses in the Gestalt completion test (Street, 1931), such that participants who reported being tired and happy generally had the greatest conceptual breadth. The present results also support the idea that low arousal positive affect promotes a broad, coarse processing style (e.g., Vanlessen, 2013; 2014).

But why might low arousal positive affect serve to promote cognitive breadth? Middlewood et al. (2016) examined whether acquiescence and sensation-seeking could explain the relationship between the tired and happy interaction and the acceptance of atypical ideas, but they did not find support for these as mediators of the relationship. In contrast, we suggest that low arousal positive affect allows one to experience greater conceptual breadth without the constraints of cognitive control. Previous reviews have already emphasized that naturally occurring positive affect and positive affect induced by rewards can modulate cognitive control (see Chiew & Braver, 2011; Pourtois et al., 2017). Being low in arousal (e.g., tired or relaxed) may also mean that cognitive control also functions less stringently. Indeed, van der Linden et al. (2003) provided evidence for reduced executive control under conditions of mental fatigue. Furthermore, van der Linden and Eling (2006) showed impaired local processing under conditions of fatigue, which was attributed to reduced focusing due to weakened attentional control. These ideas are consistent with those of Paul et al. (2021) who posit that positive affect that is low in approach motivation promotes increased breadth, distractibility, and flexibility, while positive affect high in approach motivation promotes reduced breadth and increased focus and stability. So, if broad exploration of the external environment is maximized under conditions of positive affect and low cognitive control, as posited by Vanlessen et al. (2016), then being positive and low in arousal may be an optimal combination for cognitive breadth.

Indeed, when examining the nature of the items endorsed by those who were experiencing combinations of high/low valence and high/low arousal, it became clear that the negative valence-low arousal individuals were not typically sad (the mood state typically induced for this cell), but tired and bored instead. Gable and Harmon-Jones (2010b) have shown that sadness is associated with increased breadth. However, while sadness may promote cognitive breadth, boredom may not. Indeed, some researchers have suggested that although boredom is low in arousal, it is high in motivational intensity as one tries to alleviate the boredom by actively approaching (or wishing they could approach) stimuli to alleviate the boredom (e.g., Carroll et al., 2010; Gasper & Middlewood, 2014).

Future investigations will be needed to better understand how conceptual breadth varies as a function of affective states and cognitive control. Regardless, examining conceptual breadth in relation to individual differences in affective experiences encompassing a range of valence and arousal levels demonstrates that it is the *interaction* of valence and arousal that is critical to understanding the relationship between affect and conceptual breadth and that both dimensions need to be examined in combination.

### Individual differences in naturally occurring affect

Rather than inducing a specific mood state as in previous studies, we measured individual differences in participants’ self-reported naturally occurring affect valence and arousal. In so doing, we were able to replicate some effects observed with mood inductions such as video clips or emotional pictures—for example, showing that greater positive affect is linked with greater conceptual breadth for those who are low in arousal (e.g., Fredrickson & Branigan, 2005). Furthermore, the use of the Circumplex Affect Questionnaire allowed us to examine the possible interaction between valence and arousal. Also, because affect predicts the breadth of cognition *without* induction cues here, it suggests that it may be the affect experience itself that promotes cognitive performance, not just the priming by the cues themselves. Thus, the present findings suggest that an individual’s cognitive breadth is not just linked with induced mood or momentary situational factors influencing state affect, but that individuals also bring with them their own inherent dispositions and that such individual differences are relevant to understanding differences in their perceptions and interactions in the world. Indeed, as described by Bar (2009), there is likely a reciprocal relationship between affect and breadth of associations where how one processes information (in terms of broad flexible associations versus narrow ruminations) has implications for mood, which in turn influences breadth of associations. In support of this proposal, the present results demonstrate that individual differences in naturally occurring affect can predict differences in conceptual breadth among participants who were not selected for their emotionality.

As mentioned above, participants in our sample reported a wide range of arousal, but few were very negative in valence meaning that − 1 standard deviation for valence represents neutral, not negative, affect. Therefore, the valence dimension here generally ranges from neutral to positive as opposed to ranging from negative to positive. This is quite typical for studies using naturally occurring affect measures (e.g., MacLean et al., 2010; Vermuelen, 2010) given that participants do not often choose to participate in studies during times of emotional crisis. Therefore, it should be noted that the present data do not allow us to make claims about what occurs under conditions of, or among individuals characterized by, heightened negative affect. However, similar to work highlighting the importance of individuals differences in positive affect (e.g., Paul et al., 2021), the present work offers insight as to how the relative degree of positive affect is important for conceptual breadth when coupled with arousal. Future work would benefit from examining conceptual breadth in clinical samples where negative affect is more prevalent (e.g., depression and anxiety disorders).

Finding that individual differences in naturally occurring measures of affective experience can predict conceptual breadth is consistent with the possibility that affect may underlie previously observed trait-like consistency in cognitive breadth more generally, including global–local bias (Dale & Arnell, 2013). Indeed, we speculate that the link between naturally occurring affect and cognitive breadth may explain why affect predicts performance on separate cognitive tasks in the absence of any mood inductions (e.g., MacLean et al., 2010; Vermeulen, 2010). Such speculations highlight the importance of future research testing such notions directly, for example by evaluating conceptual (or cognitive) breadth as a mediator between affective experience and performance on various other cognitive tasks such as the attentional blink.

The results here are described in terms of naturally occurring affect predicting conceptual breadth. However, it should be noted that our findings could reflect both state and/or trait affect. When giving both state and trait measures of affect in some studies we have noticed that there is typically a moderate correlation between the two. This could suggest that participants are likely to arrive in the lab in a mood state that is typical for them, and/or suggest that one might judge what is typical for them based on how they feel currently. For example, the relatively low arousal dimension scores seen here with typical undergraduate students (who one might expect to be more activated) could suggest they were using their current level of boredom or tiredness during the task when reporting their typical levels. Regardless, dispositional associations might be expected due to current states to some degree. In summary, we are less concerned with whether the present results are driven by state or trait affect, and instead emphasize that the present results clearly show that individual differences in naturally occurring affect can predict conceptual breadth.

## Conclusions

In summary, individual differences in conceptual breadth were predicted by the interaction of self-reported naturally occurring valence and arousal. Participants reporting lower arousal showed a significant positive relationship between positive affect and conceptual breadth. In contrast, participants higher in arousal showed a significant negative relationship between positive affect and conceptual breadth. The present results showing an interaction of valence and arousal on cognitive breadth can help explain previous inconsistencies in the literature about whether positive affect is associated with increases or decreases in cognitive breadth. In contrast to existing theories of cognitive breadth that highlight the importance of valence alone, or arousal alone, the present results suggest that the combination of arousal and valence is key to predicting conceptual breadth. Specifically, positive mood states may predict greater conceptual breadth in the presence of low arousal, perhaps due to a relaxation of cognitive control. The present results suggest that individual differences in affective dispositions may bias perceptions, thoughts, and behaviors and, in turn, may be biased by them.

## Supplementary Information

**Additional file 1**.** Fig. S1**. Model input specifications from the measurement model for the latent cognitive breadth factor.** Fig. S2**. Model input specifications from the structural equation model predicting the latent cognitive breadth factor using affect valence, arousal, and their interaction.

## Data Availability

Data will be publicly available on the OSF portal's data repository.

## Abbreviations

AUT: Alternative Uses Task
CFI: Comparative fit index
OCT: Object Categorization Task
RAT: Remote Associates Test
RMSEA: Root-mean-square error of approximation
SD: Standard deviation
SRMR: Standardized root-mean-square residual
