# Supplementary material for: Individual differences in naturally occurring affect predict conceptual breadth: evidence for the importance of arousal by valence interactions
Source: Cogn Res Princ Implic. 2022 Nov 18;7:98. doi: 10.1186/s41235-022-00447-w (PMC9674818; doi:10.1186/s41235-022-00447-w)
Supplement: Supplementary file 1 — Additional file 1. Fig. S1. Model input specifications from the measurement model for the latent cognitive breadth factor. Fig. S2. Model input specifications from the structural equation model predicting the latent cognitive breadth factor using affect valence, arousal, and their interaction. [file 41235_2022_447_MOESM1_ESM.docx]

**Additional file 1: Fig. S1**

Model input specifications from the measurement model for the latent cognitive breadth factor.

Note. The large oval represents the latent conceptual breadth factor; small ovals represent residual error variances. Rectangles are measured variables. Flexibility = Alternative Uses Task; Categorization = Object Categorization Task; RAT = Remote Associates Test. # = estimated model parameter.

**Additional file 1: Fig. S2**

Model input specifications from the structural equation model predicting the latent cognitive breadth factor using affect valence, arousal, and their interaction.

Note. The large oval represents the latent conceptual breadth factor; small ovals represent residual error variances or the regression residual (disturbance). Rectangles are measured variables. Flexibility = Alternative Uses Task; Categorization = Object Categorization Task; RAT = Remote Associates Test. # = estimated model parameter.
